# Emergency peripartum hysterectomy in a tertiary hospital in southern Nigeria

**DOI:** 10.11604/pamj.2013.15.60.1879

**Published:** 2013-06-20

**Authors:** Aniekan Monday Abasiattai, Aniefiok Jackson Umoiyoho, Ntiense Maurice Utuk, Emmanuel Columba Inyang-Etoh, Otobong Peter Asuquo

**Affiliations:** 1 Department of Obstetrics/Gynaecology, University of Uyo Teaching Hospital, Nigeria

**Keywords:** Emergency peripartum hysterectomy, extensive uterine rupture, uncontrollable haemorrhage, Uyo

## Abstract

**Introduction:**

Emergency peripartum hysterectomy, a maker of severe maternal morbidity and near miss mortality is an inevitable surgical intervention to save a woman's life when uncontrollable obstetric haemorrhage complicates delivery. This study was conducted in order to determine the incidence, types, indications and maternal complications of emergency peripartum hysterectomy at the University of Uyo Teaching Hospital, Uyo, Nigeria.

**Methods:**

The case records of all women who underwent emergency peripartum hysterectomy between 1^st^ January 2004 and 31^st^ December 2011 were studied.

**Results:**

There were 12,298 deliveries during the study period and 28 emergency peripartum hysterectomies were performed resulting in a rate of 0.2% or 1 in 439 deliveries. The modal age group of the patients was 26-30 years (35.7%), majority were of low parity (64.4%), while 17.9% attained tertiary level education. Half of the patients (50.0%) were unbooked while 14.3% were antenatal clinic defaulters. Extensive uterine rupture (67.8%) was the most common indication for emergency hysterectomy distantly followed by uterine atony with uncontrollable haemorrhage (17.9%). Subtotal abdominal hysterectomy was performed in 92.8% of the cases. The case fatality rate was 14.3% while the perinatal mortality rate was 64.3%.

**Conclusion:**

Emergency peripartum hysterectomy is not uncommonly performed in our centre and extensive uterine rupture from prolonged obstructed labour is the most common indication. In addition, it is associated with significant maternal and perinatal mortality. There is need to enlighten women in our communities on the benefits of ANC and hospital delivery as well as the dangers of delivering without skilled attendance. Government should consider enacting legislation to discourage people or organisations who operate unlicensed maternity homes in our environment.

## Introduction

Emergency peripartum hysterectomy (EPH) has been described as the most dramatic operation in modern obstetric practice and a marker of severe maternal morbidity and near miss mortality [1]. The procedure is usually performed when all conservative measures have failed to achieve haemostasis during life threatening obstetric haemorrhage [2].

The decision to perform an emergency hysterectomy on a young woman especially one with low parity poses a dilemma for the obstetrician particularly in our society where large family size is the norm and the premium placed on child bearing is very high [3]. However, timely decision for this surgical intervention may make the difference between life and death for the mother and greatly improve outcome.

Though the first operation of caesarean hysterectomy was originally proposed in 1768 by Joseph Cavallini in Florence [4], the first successful Obstetric hysterectomy was carried out in 1876 by Eduardo Porro from Pavia, Italy [5]. Currently, the incidence of peripartum hysterectomy is reportedly rising all over the world [2].

In the developed world the increase in the incidence of obstetric hysterectomy has been attributed to the increasing caesarean section rates, the concomitant rise in the incidence of placenta praevia and morbidly adherent placenta, and the increase in multiple pregnancy rates associated with assisted reproductive technology [2]. On the other hand, poverty, poor transportation facilities, erroneous cultural and religious beliefs, high incidence of unbooked pregnancies and poorly supervised deliveries as well as inadequate distribution of available health facilities have all made their contributions to the higher incidence of obstetric hysterectomy in the developing countries [2].

Although emergency peripartum hysterectomy is usually performed to save the life of the mother, it can be associated with maternal mortalit1y and also morbidity due to uncontrollable haemorrhage, delay in intervention, risks from blood transfusions, infection and disseminated intravascular coagulation particularly in the developing countries [2]. There is paucity of published work on emergency peripartum hysterectomy in Southern Nigeria and no study has been conducted in our centre since it was established. This study was conducted at the University of Uyo Teaching Hospital (UUTH) in order to determine the incidence, types and the indications for peripartum hysterectomy in the centre as well as the maternal outcome following the operation.

## Methods

This study was conducted at the maternity unit of the University of Uyo Teaching Hospital located in Uyo, the capital of Akwa Ibom State which is in the South-South geopolitical zone of Nigeria. The hospital has a 360-bed capacity and is the only hospital offering tertiary health care services to the people of the state with a population of about 4 million. It also serves as a referral centre for primary and secondary health facilities within the state and its environs.

The registration numbers of all women who underwent emergency peripartum hysterectomy between 1^st^ January 2004 and 31^st^ December 2011 were indentified. With the numbers, the case notes were retrieved from the medical records department for in-depth study. Information abstracted included the socio-demographic characteristics of the patients, indications for the hysterectomy, type of hysterectomy performed, cadre of surgeon, booking status of patients, mode of delivery, gestational age at delivery and maternal outcome. The data were analysed using simple proportion, rates and tables.

## Results

During the period of study there were 12,298 deliveries and 28 emergency peripartum hysterectomies were performed resulting in a rate of 0.2% or 1 in 439 deliveries. The patients? ages ranged from 18-48 years with majority (35.7%) belonging to the 26-30 years age group. Most of the patients (64.3%) were of low parity i.e. Para 1 or 2, while 7.1% of them were grandmultiparous. All the patients were Christians and married while only 17.9% attained tertiary level education. Traders (35.7%), civil servants (17.9%) and seamstress (14.3%) constituted 67.9% of the patients (Table 1).

**Table 1:** Socio-demographic characteristics of the patients. N = 28

Variable	No (%)
Age group	
≤ 20	3 (10.7)
21-25	8 (28.6)
26-30	10 (35.7)
31-35	4 (14.3)
36-40	2 (7.1)
> 40	1 (3.6)
Parity	
P1	6 (21.4)
P2	12 (42.9)
P3	4 (14.3)
P4	3 (10.7)
≥ P5	2 (7.1)
Educational status	
Primary level	7 (25.0)
Secondary level	13 (46.4)
Tertiary level	5 (17.9)
Not recorded	3 (10.7)
Occupation	
Trader	10 (35.7)
Civil servant	5 (17.9)
Seamstress	4 (14.3)
Housewife	3 (10.7)
Farmer	2 (7.1)
Student	2 (7.1)
Teacher	1 (3.6)
Hair dresser	1 (3.6)

Fourteen patients (50%) were unbooked and hence had no formal antenatal care. Four booked patients (14.3%) defaulted and were brought from unorthodox health facilities only after complications had occurred. All the unbooked patients and half of the antenatal clinic defaulters were brought from spiritual churches where they attempted to deliver. Booked patients accounted for 28.6% of the study population. The major indication for emergency peripartum hysterectomy was extensive uterine rupture (67.8%) and in all cases the fetuses had been extruded into the peritoneal cavity. Other indications were morbidly adherent placenta and placenta praevia which accounted for 14.3% each respectively (Table 2).

**Table 2:** Booking status of the patients and indications for hysterectomy

Variable	No (%)
Booking status	
Unbooked	14 (50.0)
Booked	8 (28.6)
Defaulted	4 (14.3)
Referred	2 (7.1)
Indication for emergency hysterectomy	
Extensive uterine rupture	19 (67.8)
Uterine atony with uncontrollable postpartum haemorrhage	5 (17.9)
Placenta praevia with uncontrollable postpartum haemorrhage	2 (7.1)
Morbidly adherent placenta	2 (7.1)

Seven patients (25.0%) were delivered by caesarean section, 7.1% of the patients had spontaneous vaginal delivery (SVD) while 67.8% had laparotomy. Subtotal abdominal hysterectomy was performed in 92.8% of the cases (with bladder repair in 4 cases) while total abdominal hysterectomy was performed in 7.1% of the cases. Consultants performed 64.3% of the hysterectomies while senior registrars performed 34.7% of the procedures.

The maternal complications are shown in Table 3. The most common maternal complication was anaemia (32.1%). There were four maternal deaths resulting in a case fatality rate of 14.3%. There were 18 (64.3%) perinatal deaths (15 fresh still births and 4 macerated still births).

**Table 3:** Maternal complications

Complication	No (%)
Anaemia	9 (32.1)
Pelvic sepsis	4 (14.3)
Mortality	4 (14.3)
Burst abdomen	3 (10.7)
Wound sepsis	2 (7.1)
Heart failure	1 (3.6)
Pulmonary oedema	1 (3.6)
Vesico-vaginal fistula	1 (3.6)
Omental haematoma	1 (3.6)
Chest infection	1 (3.6)

## Discussion

Emergency peripartum hysterectomy remains an inevitable surgical intervention to save a woman's life when uncontrollable obstetric haemorrhage complicates delivery. The rate of EPH in our centre is similar to what obtains in other Nigerian centres [6–9], and also other centers in sub-Saharan Africa [10]. While it is lower than rates recorded from studies conducted in Pakistan and India in South Central Asia which vary from 0.4% - 0.7% [2, 11, 12], the EPH rate is much higher than those reported from Saudi Arabia (1 in 2,559 deliveries) [1] and the developed countries of America and Europe where the incidence of EPH is approximately 1 in 2000 deliveries [13]. A population based study in Canada revealed an incidence of 0.53 per 1000 deliveries while another study in the United Kingdom revealed a national incidence for peripartum hysterectomy of approximately 1 per 2,500 births [13].

The relatively high incidence of EPH in our environment and other developing countries may not be unconnected with the inadequate obstetric services prevalent in these areas and the large numbers of unbooked emergencies and antenatal clinic defaulters as demonstrated by this study. Several studies from developing countries including Nigeria have shown poor maternal and perinatal outcome in women who fail to obtain antenatal care and those who default and are only brought to hospital when obstetric complications occur [10, 14, 15].

In this study most of the patients were in the peak period of their reproductive life as most of them were between 26-30years of age and were of low parity (Para 1& 2). Interestingly, this is in direct contrast to what obtained in other centres [6, 10, 12, 16] where most of the patients in those series were above thirty years of age and were grandmultiparous hence almost at the end of their reproductive carriers. Sadly, this goes to highlight the adverse reproductive health effects removal of the uterus will have on these patients particularly in an environment like ours where failure of menstruation is perceived by women to be tantamount to loss of their self image and the premium placed on childbearing is very high. The uterus is an organ highly treasured and hysterectomy is not a widely accepted operation [17]; hence loss of reproductive potential at a relatively early age can result in very devastating consequences including marital disharmony, divorce and even psychological disorders [18].

The most common indication for peripartum hysterectomy in this study was extensive uterine rupture. This is similar to findings from other centres in Nigeria [3, 7, 8, 9] and other developing countries [2, 11, 12], but varies from results from developed countries were placenta praevia and morbidly adherent placenta are the most frequent indications [13, 19]. Interestingly, in contrast to what obtains in Asia where injudicious use of oxytocics or obstetric manipulation were the main causes of uterine rupture [2, 11, 12], in our review, all the cases of uterine rupture followed prolonged obstructed labour. This goes to highlight the significant contribution ruptured uterus from prolonged obstructed labour makes to poor reproductive health indices of our women and also to the alarming high maternal mortality and morbidity in our environment.

All the patients were brought from spiritual churches where they had laboured for over 48 to 72 hours, in moribund states with extensive ruptures, torn friable uteruses, sometimes gangrenous and their fetuses completely extruded from the uterus and lying freeing in the peritoneal cavity. In south-south Nigeria, spiritual churches engage in maternity services even though they lack the facilities and their personnel lack the needed skill and expertise to conduct deliveries safely [20]. Curiously, when they are faced with dire complications, rather than refer patients to licensed maternity centres, they resort to faith-based interventions like prayers and worship [21]. The women are also prophetically warned to avoid delivery in hospital in order to avoid untimely death from the devil and wicked people [20].This scenario makes it impossible for the women to seek help early in licensed health facilities following complications.

Sub-total abdominal hysterectomy was the most commonly performed surgical procedure in our review as also documented in some other studies [2, 3, 7, 10, 22]. This appears to be the procedure of choice to control uncontrollable obstetric haemorrhage in affected patients as it is relatively easier and quicker to perform. In addition, in patients with extensive uterine rupture it is associated with less post operative morbidity since the infected and torn/ragged uterus is removed [3].

The maternal mortality rate of 14.3% in this study may have resulted largely from moribund cases who presented late to hospital, thus leaving no time for maternal salvage. This mortality is however lower than the 59.1%, 20.0% and 19.3% that were obtained in Zaria [3], Calabar [9], and Lagos [7]. The reason for this disparity has not been adduced by this study; however, it is probably that the policy of aggressive resuscitation that was practiced in our hospital during the period of this study and the early recourse to hysterectomy could have resulted in the difference. Notwithstanding, when compared to what obtains in the developed world where several series of peripartum hysterectomies are reported without any maternal deaths [23], our figures are rather high.

The PNMR of 64.3% following EPH was similar to findings from other developing countries including Nigerian [3, 7, 9, 24].This may have been influenced by the most common indication for EPH in this series, which was extensive uterine rupture with its devastating consequences on the foetus. The most frequent complications were postoperative anaemia and postoperative pelvic sepsis. These complications probably reflect the state of the patients preoperatively with regard to exsanguinations, presence of sepsis and inadequate intrapartum supervision.

## Conclusion

EPH is not uncommonly performed in our centre and extensive uterine rupture from prolonged obstructed labour is the most common indication. In addition, it is associated with significant maternal and perinatal mortality. There is need to enlighten women in our communities on the benefits of ANC and hospital delivery as well as the dangers of delivering without skilled attendance. Government should consider enacting legislation to discourage people or organisations who operate unlicensed maternity homes in our environment.
